# Molecular subtyping of acute myeloid leukemia through ferroptosis signatures predicts prognosis and deciphers the immune microenvironment

**DOI:** 10.3389/fcell.2023.1207642

**Published:** 2023-08-24

**Authors:** Denggang Fu, Biyu Zhang, Shiyong Wu, Jueping Feng, Hua Jiang

**Affiliations:** ^1^ College of Medicine, Medical University of South Carolina, Charleston, SC, United States; ^2^ School of Chemical Engineering and Pharmacy, Wuhan Institute of Technology Wuhan, Wuhan, China; ^3^ Department of Pediatrics, The Wells Center for Pediatric Research, Indiana University School of Medicine, Indianapolis, IN, United States; ^4^ Wuhan Fourth Hospital, Wuhan, Hubei, China; ^5^ Department of Radiation Oncology, School of Medicine, Stanford University, San Francisco, CA, United States

**Keywords:** acute myeloid leukemia, ferroptosis, molecule subtyping, outcome, immune microenvironment

## Abstract

Acute myeloid leukemia (AML) is one of the most aggressive hematological malignancies with a low 5-year survival rate and high rate of relapse. Developing more efficient therapies is an urgent need for AML treatment. Accumulating evidence showed that ferroptosis, an iron-dependent form of programmed cell death, is closely correlated with cancer initiation and clinical outcome through reshaping the tumor microenvironment. However, understanding of AML heterogeneity based on extensive profiling of ferroptosis signatures remains to be investigated yet. Herein, five independent AML transcriptomic datasets (TCGA-AML, GSE37642, GSE12417, GSE10358, and GSE106291) were obtained from the GEO and TCGA databases. Then, we identified two ferroptosis-related molecular subtypes (C1 and C2) with distinct prognosis and tumor immune microenvironment (TIME) by consensus clustering. Patients in the C1 subtype were associated with favorable clinical outcomes and increased cytotoxic immune cell infiltration, including CD8^+^/central memory T cells, natural killer (NK) cells, and non-regulatory CD4^+^ T cells while showing decreased suppressive immune subsets such as M2 macrophages, neutrophils, and monocytes. Functional enrichment analysis of differentially expressed genes (DEGs) implied that cell activation involved in immune response, leukocyte cell–cell adhesion and migration, and cytokine production were the main biological processes. Phagosome, antigen processing and presentation, cytokine–cytokine receptor interaction, B-cell receptor, and chemokine were identified as the major pathways. To seize the distinct landscape in C1 vs. C2 subtypes, a 5-gene prognostic signature (LSP1, IL1R2, MPO, CRIP1, and SLC24A3) was developed using LASSO Cox stepwise regression analysis and further validated in independent AML cohorts. Patients were divided into high- and low-risk groups, and decreased survival rates were observed in high- vs. low-risk groups. The TIME between high- and low-risk groups has a similar scenery in C1 vs. C2 subtypes. Single-cell-level analysis verified that LSP1 and CRIP1 were upregulated in AML and exhausted CD8^+^ T cells. Dual targeting of these two markers might present a promising immunotherapeutic for AML. In addition, potential effective chemical drugs for AML were predicted. Thus, we concluded that molecular subtyping using ferroptosis signatures could characterize the TIME and provide implications for monitoring clinical outcomes and predicting novel therapies.

## Introduction

Acute myeloid leukemia (AML) is a type of aggressive blood malignancy characterized by the stagnant transition of immature myeloid cells to mature cells in the bone marrow and peripheral blood (Thomas and Majeti, 2017). Despite great advancements in therapies for most blood cancer types, treatment options for AML are still limited over decades. Therapeutic resistance and a high rate of relapse lead to a low 5-year survival rate (Jemal et al., 2017; Marando and Huntly, 2020). Chemotherapy has been used as standard care for patients with AML; unfortunately, most patients eventually succumb to this disease due to relapse or resistance. New therapies such as molecular targeting therapy, bispecific antibodies, chimeric antigen receptor (CAR) T-cell therapy, and immune checkpoint inhibitors (ICIs) are emerging, aiming for leaps forward (Winer and Stone, 2019; Marofi et al., 2021; Nair et al., 2021). For example, targeting CD33 on AML cells using gemtuzumab ozogamicin showed remission in AML patients, while serious side effects such as severe myelosuppression and tumor lysis syndrome limited its use in clinical practice (Feldman et al., 2005). CAR T-cell and bispecific antibody therapies (He et al., 2020) are still under pre-clinical or clinical testing stages, and patients still face serious complications such as graft-versus-host disease and cytokine release syndrome (Wang et al., 2019a). Identification of novel targets or less toxic and more efficient therapy strategies is an urgent need.

Ferroptosis was recognized as a non-apoptotic and iron-dependent lipid peroxidation-induced type of programmed cell death, which is distinct from other forms of cell deaths, including necroptosis, apoptosis, and autophagy, in morphology and mechanisms (Dixon et al., 2012; Stockwell et al., 2017). The hallmarks of ferroptosis are the redox-active iron levels, dysfunction of lipid peroxide repair capacity, and oxidation of polyunsaturated fatty acid. Accumulating evidence indicated that ferroptosis susceptibility is mediated by RAS/MAPK signaling, amino acid and iron metabolism, cell adhesion, phospholipid biosynthesis, p53 mutant status, and NRF2 activity (Li J. et al., 2020). An increasing number of genes, such as G6PD, TP53 (Jiang et al., 2015), GPX4 (Ma et al., 2016), SLC7A11 (Wang et al., 2016), and DHODH (Mao et al., 2021), have been identified as drivers, regulators, and suppressors of ferroptosis which are called ferroptosis-associated genes (FAGs) in tumor cells. It is found that ferroptosis of tumor cells or immune cells is correlated with cancer progression and treatment response and plays multiple roles in biological regulations and signaling pathways (Stockwell et al., 2017; Shen et al., 2018; Stockwell and Jiang, 2019). Previous studies have demonstrated that ferroptosis acts as a promising anticancer therapeutic strategy (Yang et al., 2014; Miess et al., 2018; Badgley et al., 2020). APR-246, which targets p53-mutated proteins, can induce ferroptosis in AML, representing a new therapeutic drug (Birsen et al., 2021). 4-Amino-2-trifluoromethyl-phenyl retinate (ATPR) is a novel all-trans retinoic acid derivative which exhibits strong anticancer activity in AML. Targeting ferroptosis promotes ATPR-induced AML differentiation via the ROS–autophagy–lysosomal pathway (Du et al., 2020). Zhu et al. (2019) showed that typhaneoside can prevent AML progression by suppressing proliferation and inducing ferroptosis associated with autophagy. Furthermore, Wang et al. (2019b) found that ferroptosis mediates antitumor activities; for example, immunotherapy-activated CD8^+^ T cells by the combination of PD-1 blockade and CTLA-4 therapy aggravate ferroptosis-specific lipid peroxidation in tumor cells, suggesting that enhanced ferroptosis contributes to immunotherapy efficacy. Most studies on FAGs focused on solid tumors, but their functions in AML were not well understood. Therefore, it might provide new prospects for developing anti-leukemia agents for treating AML through the understanding of the ferroptosis regulatory heterogeneity of the tumor microenvironment (TME).

In this study, we systematically profiled FAGs expression datasets and clinical features in patients with AML obtained from The Cancer Genome Atlas (TCGA) and GEO (GSE37642, GSE12417, GSE31580, and GSE106291) databases. The molecular diversity of AML was delineated by molecular subtyping using ferroptosis signatures. The tumor immune microenvironment (TIME) was characterized using multiple immune cell subset deconvolutions, and the immune status between classifications was compared. To further specify the TIME, we developed a prognostic signature based on the overall survival (OS)-related differentially expressed genes between molecular subtypes. External validation demonstrated that the signature could predict patient prognosis and reflect TIME, which might have implications for developing new therapies by targeting ferroptosis in AML.

## Methods and materials

### AML gene expression datasets and processing

An AML gene expression dataset with clinical features was downloaded from The Cancer Genome Atlas (TCGA) database and regarded as the training set. The gene expression profile of 514 normal cases was acquired from the Genotype-Tissue Expression (GTEx) database. For external validation sets, five AML cohorts with clinical information (GSE37642, GSE12417, GSE10358, and GSE106291; Supplementary Table S1) were downloaded from Gene Expression Omnibus (GEO). Briefly, raw “CEL” files were downloaded. Those samples measured by the same platform were merged following a robust multi-array averaging method using affy packages for background adjustment and quantile normalization, and batch effects removal was performed by the combat algorithm in the sva package (Leek et al., 2012). The samples measured using the GPL96 platform from GSE37642 and GSE12417 were merged as validation set 1. The samples measured using the GPL570 platform from GSE37642, GSE12417, and GSE10358 were combined as validation set 2. The GSE106291 dataset was used as validation set 3.

### Differential gene expression analysis

Differential gene expression analysis was conducted by comparing GTEx normal blood samples and AML samples using the limma package (Ritchie et al., 2015) in the R platform. Differentially expressed genes (DEGs) were determined with the cutoff value of |LogFC| >1 and adjusted *p* < 0.05.

A total of 259 ferroptosis-related genes (FAGs), including drivers, suppressors, and markers, were obtained from the FerrDb database (http://www.zhounan.org/ferrdb/) (Zhou and Ferrdb, 2020). Differentially expressed FAGs were selected for further analysis.

In addition, protein–protein interaction network analysis of differentially expressed FAGs was retrieved from the STRING database and illustrated using Cytoscape (version 3.8).

### Functional enrichment analysis

To delineate the difference in enriched biological activities and signaling pathways of the target gene list, Gene Ontology (GO), including biological processes (BP), molecular function (MF), and cellular components (CC), was conducted using the ‘‘clusterProfiler’’ package (Wu T. et al., 2021). In addition, the enriched pathways were identified through Kyoto Encyclopedia of Genes and Genomes (KEGG) enrichment analysis. A *p*-value and a q-value <0.05 were considered statistically significant.

### Identification of overall survival (OS)-related FAGs

The expression matrix of these differentially expressed FAGs was extracted from the TCGA-AML dataset, and the clinical features were merged with the expression matrix. OS-related FAGs in AML with *p* < 0.05 were identified through univariate Cox regression analysis.

### Molecular subtyping of AML using consensus clustering

To assess the molecular heterogeneity within the AML dataset related to FAGs, cluster robustness was calculated through K-means consensus clustering using the ConsensusClusterPlus package (Wilkerson and Hayes, 2010), with cluster numbers from 2 to 10. The minimum number of stable clusters was determined by plotting the cumulative distribution.

Differential gene expression analysis was conducted to screen the differentially expressed genes between molecular subtypes, and functional enrichment analysis was used to investigate the biological processes and pathways as described previously.

### Characterization of the tumor immune microenvironment (TIME) between ferroptosis subtypes

The tumor and its surrounding microenvironment, including immune cells, stromal cells, signaling molecules, and extracellular matrix, constitute the tumoral niche. The TIME between ferroptosis-related subtypes was deciphered from transcriptomes using multiple cell subset deconvolution algorithms including CIBERSORT (Newman et al., 2019), TIMER (Li T. et al., 2020), and xCell (Aran et al., 2017). The relative percentage of infiltrating cell subsets between the subtypes was compared using the Wilcoxon test.

In addition, expression levels of inhibitory or stimulatory checkpoint molecules between the subtypes were compared using the Wilcoxon test. A *p*-value <0.05 was considered statistically significant. The human leukocyte antigen (HLA) system is a complex of proteins that are closely involved in the regulation of immune responses such as antigen presentation and stimulation of T-helper cells (Sendker et al., 2021). The expression of these HLA molecules was also compared between the subtypes.

### Construction and validation of ferroptosis subtyping-related signature

Differentially expressed genes between subtypes were identified using the limma package with |LogFC| >1 and adjusted *p* < 0.05. OS-related DEGs were screened using univariate Cox regression analysis. To avoid the overfitting effect, OS-related DEGs were selected using the least absolute shrinkage and selection operator (LASSO) algorithm, following the multivariate Cox regression analysis. The minimum number of features that comprised the optimal signature was determined by the Akaike information criterion (AIC) (Vrieze, 2012). The ferroptosis subtype-associated signature risk score (termed as FSAscore) for each patient was formulated as follows:

$$FSAscore=\sum \begin{array}{l} n\\ i \end{array}Coef\;x\;S_{i},$$

where Coef represents the regression coefficient, “i” represents the signature gene, “S” represents the relative value of the ferroptosis signature gene expression, and “n” represents the number of signatures.

Model discrimination ability was predicted by the receiver operating characteristic (ROC) curve using the timeROC package (Blanche et al., 2013). The risk score for individual patients was calculated using the signature, and patients were divided into high- and low-risk groups by the median risk score. The performance of the signature in prognosis prediction was assessed by a log-rank test using the “survminer” package and presented using the Kaplan–Meier curve.

The prognostic utility of the signature was independently validated by three external validation sets. Furthermore, the predictive performance was assessed by the ROC curve.

### Compound sensitivity and immunotherapy response prediction

To investigate the potential of the signature in predicting treatment sensitivity, NCI-60 tumor cell line growth inhibition screening was used to identify the ferroptosis defined nature of the cell lines to the sensitivity of relevant compounds in the NCI Development Therapeutics Program (DTP) (dtp.cancer.gov). The expression profile based on Affymetrix U133 Plus 2.0 microarray was normalized using GC-robust multi-array averaging. FSAscore was derived and correlated with compound sensitivity using CellMiner (discover.nci.nih.gov/cellminer) (Shankavaram et al., 2009).

### Single-cell analysis of ferroptosis subtype-related signature

Since five DEGs that constitute the risk signature were prognostic in AML, we compared the expression of these genes in AML vs. normal cells using the GEPIA database (http://gepia.cancer-pku.cn/). Single-cell-level analysis was further conducted to verify their transcripts in the AML microenvironment in the Tumor Immune Single-cell Hub (TISCH, http://tisch.comp-genomics.org/; GSE116256 and GSE154109) (Sun et al., 2021).

## Results

### Identification of differentially expressed FAGs in AML

**FIGURE 1:**
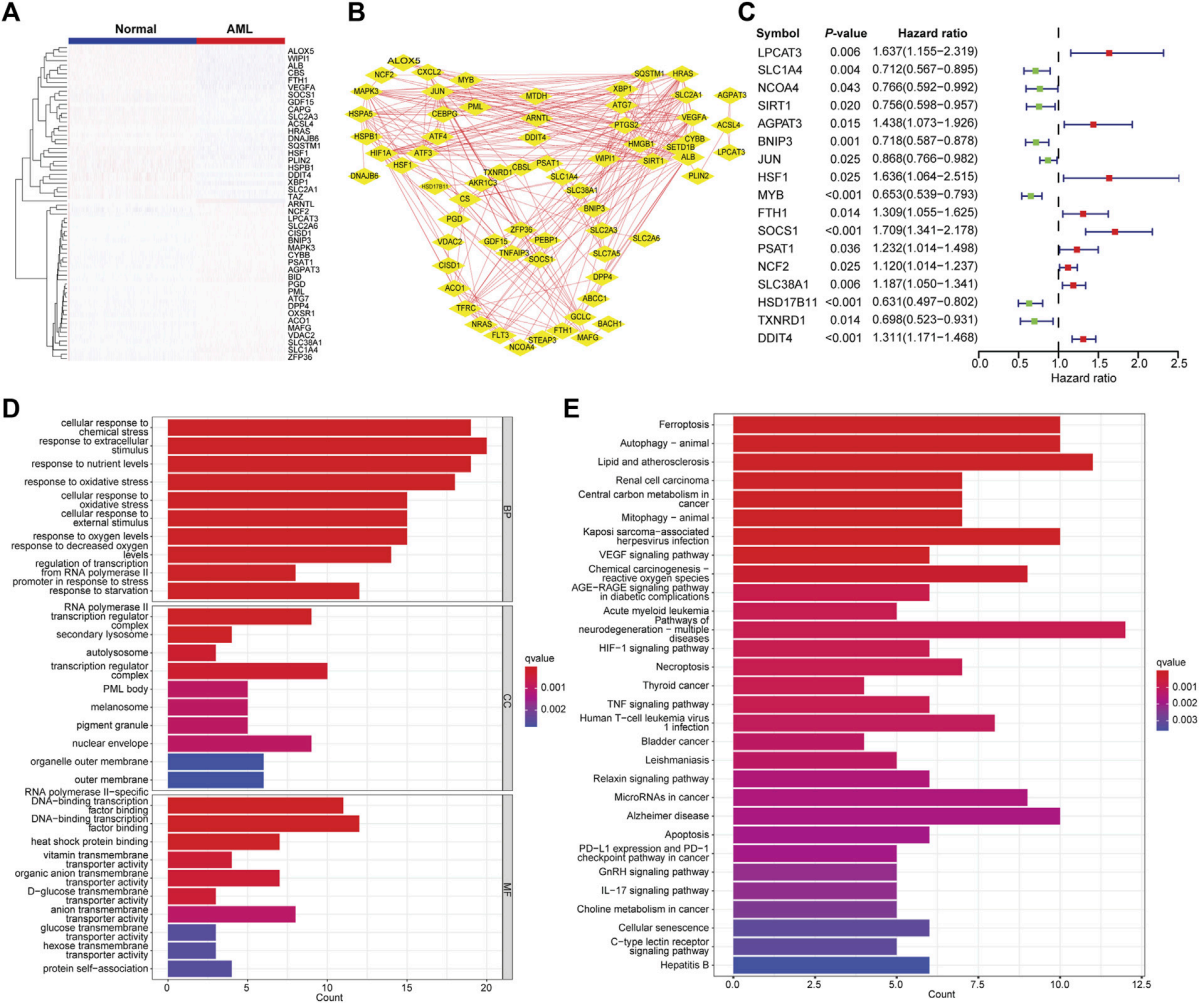
Identification of differentially expressed FAGs in AML. **(A)** Heatmap of differentially expressed FAGs in AML vs. normal samples. **(B)** The protein–protein interaction network of differentially expressed FAGs in AML. **(C)** Forest plot showing the correlations of differentially expressed FAG expression with clinical outcomes of AML patients. **(D)** Significantly enriched GO terms (biological process, molecular function, and cellular component) of differential expressed FAGs. **(E)** Significantly enriched KEGG signaling pathways of differentially expressed FAGs.

The differential gene expression analysis was performed to compare the FAG expression levels between AML and normal samples. A total of 3,093 DEGs were identified, of which 1,501 genes were upregulated, and 1,592 genes were downregulated in AML. We found 72 differentially expressed FAGs in AML vs. normal samples. These FAGs showed a distinct expression pattern (Figure 1A), which was also reflected in two well-separated dimensions calculated by principal component analysis (Supplementary Figure S1A). Protein–protein interaction network analysis was performed to investigate the potential interactions of these FAGs. Two significant modules, HIF1A and ALB, were identified in the whole network sorted by the degree value (Figure 1B). The HIF1A module contained 10 nodes and 31 edges, and the ALB module had 11 nodes and 34 edges. Most of the genes in the HIF1A module play leukemia-promoting roles in AML, such as HIF1A (Abdul-Aziz et al., 2018), CEBPG (Jiang et al., 2021), JUN (Zhou et al., 2017), and ATF4 (Heydt et al., 2018). These genes also contributed to treatment resistance in AML (Zhu et al., 2020). Genes with similar biological functions in the ALB module were found such as SIRT1 (Li et al., 2014), XBP1 (Kharabi Masouleh et al., 2015), and ATG7 (Piya et al., 2016). In addition, the top 10 targets (HIF1A, JUN, ALB, SIRT1, MAPK3, VEGFA, PTGS2, SLC2A1, HRAS, and HSPA5) with the highest degrees in the network were regarded as the hub genes using the cytoHubba plugin (Chin et al., 2014) (Supplementary Figure S1B). Inhibition of these targets represents potential therapeutics for AML, as evidenced by previous reports. The clinical relevance of differentially expressed FAGs in AML was determined, and 17 of 72 FAGs were associated with patient survival (Figure 1C). GO term analysis indicated that these FAGs are enriched in stimulus, chemical, or oxidative stress-related biological processes, lysosome and membrane, transcription factor binding, and transmembrane transporter activities (Figure 1D). Cell death events, including ferroptosis, autophagy, necroptosis, apoptosis, and cellular senescence, HIF1 signaling, acute myeloid leukemia, and metabolic pathways, such as cancer central carbon metabolism, were listed as the most enriched pathways (Figure 1E). Dysfunctions of these pathways in AML have been demonstrated to promote disease progression or induce therapy resistance (Garcia-Bates et al., 2008; Carneiro et al., 2015; Saito et al., 2015; Piya et al., 2017), which suggests that these FAGs play a non-redundant role in AML progression.

### Molecular subtyping of AML

**FIGURE 2:**
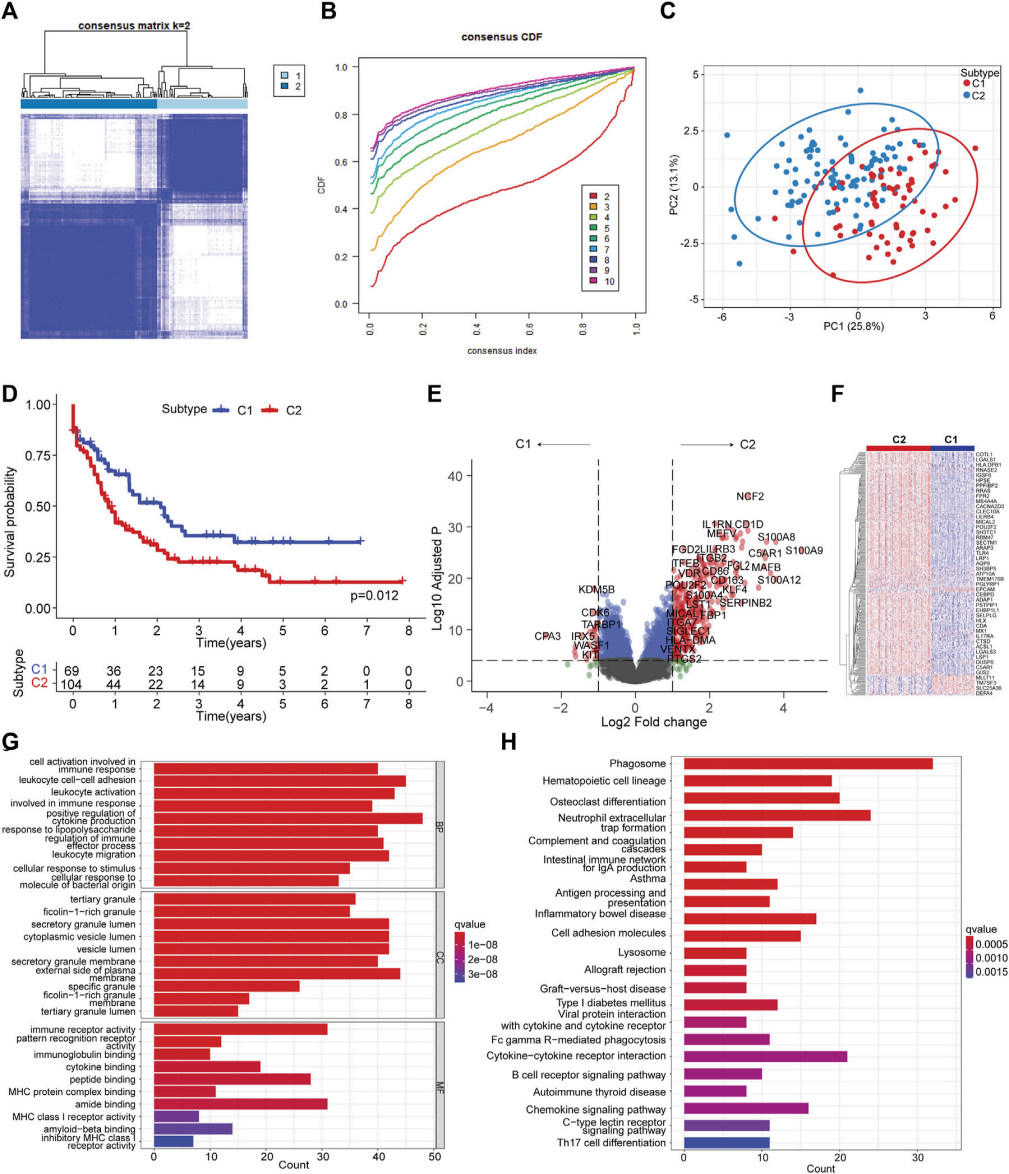
Ferroptosis molecular subtyping by consensus clustering. **(A)** Consensus clustering with k = 2. **(B)** Cumulative distribution from consensus matrices for clustering with 2–10 clusters. **(C)** Principal component analysis illustrating C1 and C2 subtypes. **(D)** Kaplan–Meier curve of C1 vs. C2 subtypes. The log-rank test was used to determine the survival difference. **(E)**. Volcano plot showing differentially expressed genes by comparing C2 vs. C1 subtypes with |LogFC| >1 and Log_10_ (adjusted *p*) < 0.05. **(F)** Heatmap of differentially expressed genes between C2 vs. C1 subtypes. **(G)** Significantly enriched GO terms of differentially expressed genes between C2 vs. C1 subtypes. **(H)** Significantly enriched KEGG pathways of differentially expressed genes between C2 vs. C1 subtypes.

To delineate the molecular heterogeneity of AML linked to ferroptosis, we attempted to determine the ferroptosis-related molecular subtypes using consensus clustering based on the transcriptome of OS-related FAGs. Two subtypes (C1 vs. C2) were identified with distinct ferroptosis gene expression patterns following K-means clustering (Figures 2A–C). Decreased OS was observed in patients in the C2 subtype compared to those in the C1 subtype (Figure 2D). A favorable clinical outcome in C1 prompted us to comprehend the potential factors such as key DEGs and signaling pathways that modulate the prognosis. Differential gene expression analysis found that many upregulated genes in the C2 subtype were associated with increased leukemia cell survival, proliferation, and drug resistance, such as S100A8, S100A9, LILRB3, KLF4, LST1, and ITGB2 (Figure 2E). In addition, the differentially expressed genes showed apparent disparity between C1 and C2 subtypes (Figure 2F). These upregulated leukemia-promoting genes might promote disease progression. To identify the difference in key pathways involved, GO enrichment analysis was conducted to identify the biological processes of DEGs. The results suggested that these DEGs were enriched in the biological processes of cell activation involved in immune response, cytokine production, and leukocyte migration (Figure 2G). Phagosome, antigen processing and presentation, cell adhesion molecules, cytokine–cytokine receptor interaction, Fc gamma R-mediated phagocytosis, B-cell receptor, and chemokine signaling pathways were the main significant pathways that closely correlate with immune response during antitumor activities (Figure 2H). Therefore, ferroptosis molecule classification might characterize the tumor immune microenvironment (TIME).

### Characterization of TIME in ferroptosis subtypes

**FIGURE 3:**
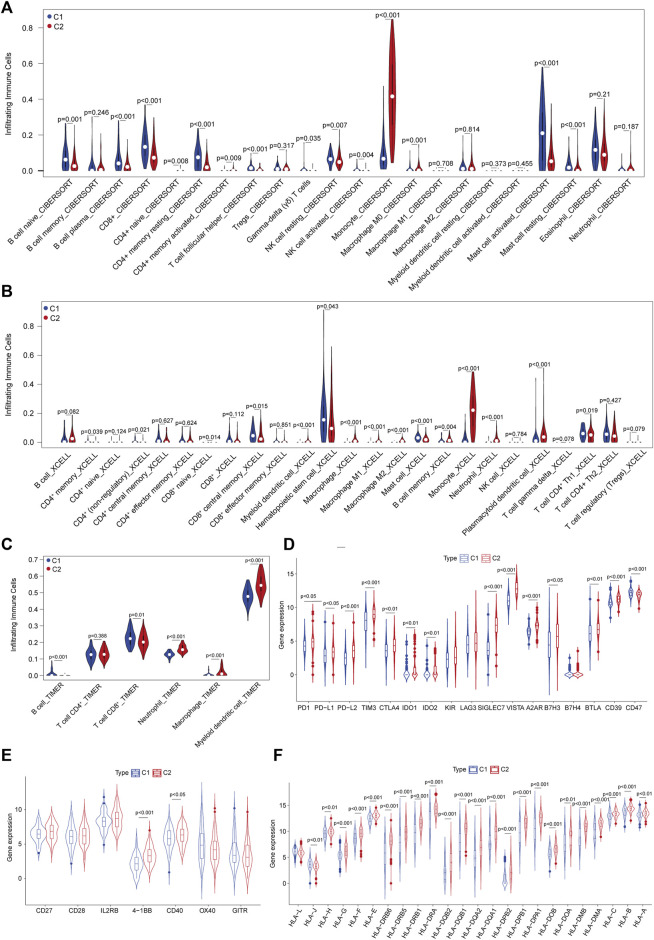
Characterization of the tumor immune microenvironment (TIME) defined by ferroptosis-related subtyping. **(A)** Abundance of infiltrated immune cell subsets in C1 vs. C2 subtypes deconvoluted using CIBERSORTx in the TCGA–AML dataset. **(B)** Abundance of infiltrated immune cell subsets in C1 vs. C2 subtypes deconvoluted using xCell. **(C)** Abundance of infiltrated immune cell subsets in C1 vs. C2 subtypes deconvoluted using TIMER. **(D)** Expression levels of inhibitory immune checkpoint molecules between C1 vs. C2 subtypes. **(E)** Expression levels of stimulatory immune checkpoint molecules between C1 vs. C2 subtypes. **(F)** Expression levels of human lymphocyte antigens between C1 vs. C2 subtypes.

We noted that these DEGs were mainly enriched in immune regulatory pathways, which prompted us to investigate the TIME between C1 and C2 subtypes. Multiple cell subset deconvolution algorithms, including CIBERSORTx, TIMER, and xCell, were used to characterize and compare various infiltrating cell types in these two subtypes. The CIBERSORTx deconvolution revealed that CD8^+^ T cells, B-cell plasma cells, NK cells, and CD4^+^ memory resting cells showed higher infiltration in patients in the C1 subtype, while monocyte, activated CD4^+^ memory cells, and macrophages showed lower infiltration in those in the C2 subtype (Figure 3A). This suggested that patients in C1 have enhanced anti-leukemia TIME compared to those patients in the C2 subtype. xCell analysis also indicated similar high infiltration of reactive immune cell subsets such as CD8^+^ effector memory cells, B cells, non-regulatory CD4^+^ T cells in the C1 subtype and low infiltration of M2 macrophages, plasmacytoid dendritic cells, and myeloid dendritic cells (Figure 3B). The results of TIMER deconvolution were consistent with the evidence of activated TIME in the C1 subtype (Figure 3C). Further assessment of the expression of immune inhibitory molecules revealed that most inhibitory checkpoints including PD-1, PD-L1/L2, TIM3, CTLA4, VISTA, BTLA, and SIGLEC7 showed significantly increased expression in C2 vs. C1 subtypes, suggesting that suppressive TIME in C2 might lead to decreased immune response (Figure 3D). The majority of stimulatory checkpoint molecule expression did not show any significant difference between these two subtypes other than 4-1BB and OX40 (Figure 3E). In addition, most of the human leukocyte antigen (HLA) genes were upregulated in C2 compared to the C1 subtype (Figure 3F). These results indicated that C2 was correlated with the dysfunctional immune niche, implying that immune checkpoint blockade might represent promising immunotherapeutics for AML patients in the C2 subtype.

### Construction and validation of ferroptosis subtype-related signature

**FIGURE 4:**
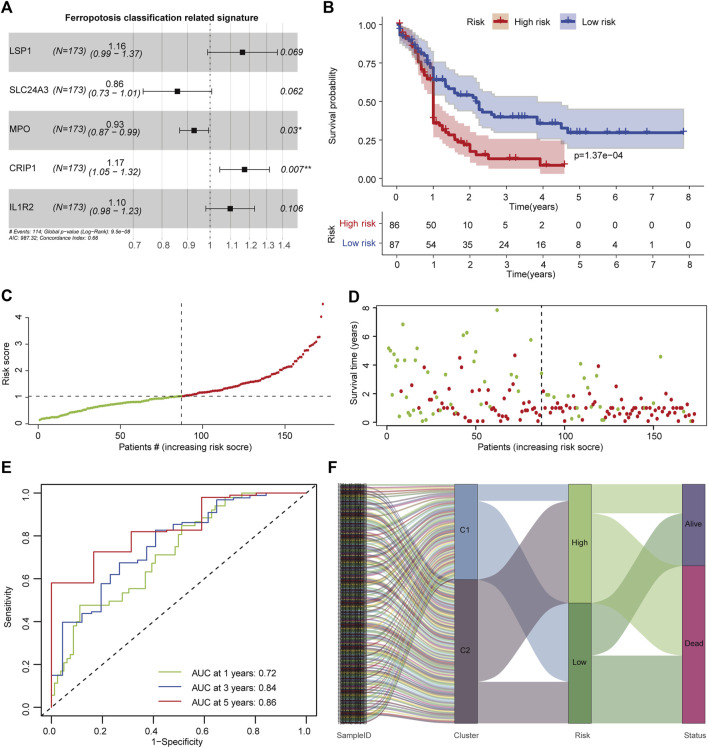
Construction of the ferroptosis-related classification-based signature. **(A)** Hazard ratio of the signature genes. **(B)** Kaplan–Meier curve of patients in high- and low-risk groups. The log-rank test was used to determine the survival difference. **(C)** Distribution of patient risk scores. **(D)** Patient survival time and risk scores. **(E)** Receiver operating characteristic (ROC) curves of the prognostic signature for 1-, 3-, and 5-year in the TCGA-AML dataset. **(F)** Alluvial diagram showing the relationship of molecular subtypes, risk groups, and survival status.

To develop a signature that could characterize the TIME difference between C1 and C2 subtypes, OS-related DEGs were determined using univariate Cox regression analysis (Supplementary Figure S2). To avoid overfitting, minimum features were selected by LASSO regression (Supplementary Figures S3A, B). A ferroptosis classification-defined prognostic signature was constructed through multivariate Cox stepwise regression analysis. The optimal signature that comprised five genes (LSP1, SLC24A3, CRIP1, MPO, and IL1R2) was determined by the Akaike information criterion (AIC) algorithm using the survival package (Figure 4A). The FSAscore was calculated using the following formula: FSAscore = LSP1 *(0.1504) + MPO * (−0.0743) + IL1R2 * (0.0934) + CRIP1 * (0.1599) + SLC24A3 * (−0.1516). Patients were divided into high- and low-risk groups based on the median FSAscore. To determine the predictive potential of the FSAscore, the Kaplan–Meier curve was generated, and decreased OS was observed in patients in the high-risk group compared to those in the low-risk group (*p* = 1.37e-4, Figure 4B). The number of deaths increased with the increasing FSAscore (Figures 4C, D). The predictive potential of the FSAscore was evaluated by the ROC curve. The AUC values of 1-, 3-, and 5-year survival of FSAscore for AML patients were 0.72, 0.84, and 0.86, respectively (Figure 4E), suggesting that FSAscore has high predictive performance. Furthermore, alluvial plots clearly showed that most of the patients in the C2 subtype have FSAscore; on the contrary, patients with low-risk FSAscore were mainly concentrated in the C1 subtype (Figure 4F).

**FIGURE 5:**
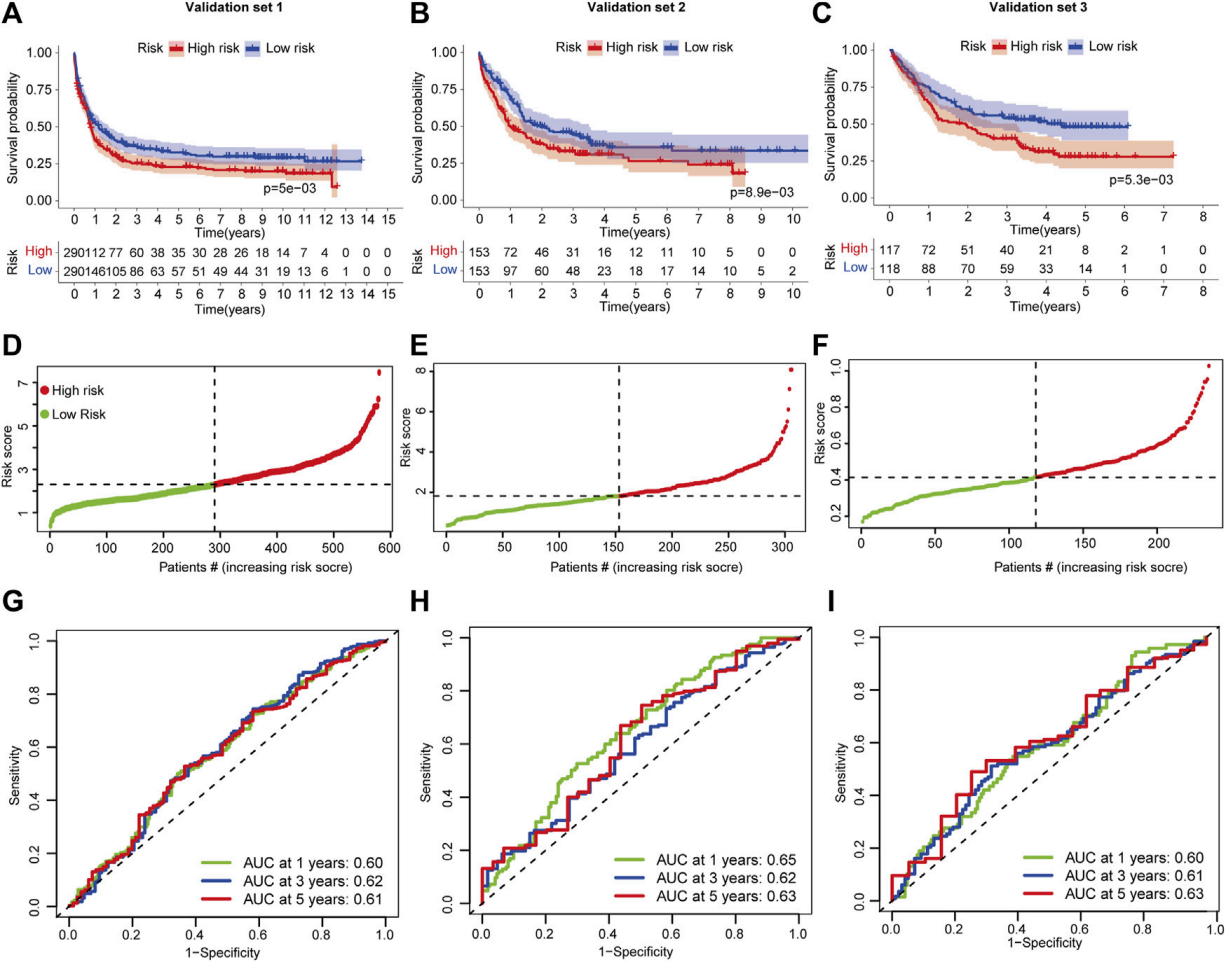
Validation of the ferroptosis-related classification-based signature. **(A–C)** Kaplan–Meier curves of patients in high- and low-risk groups in AML validation sets 1/2/3. The log-rank test was used to determine the survival difference. **(D–F)** ROC curves of the prognostic signature for 1-, 3-, and 5-year in AML validation set -1/-2/-3. **(D–F)** Distribution of patient risk scores in AML validation set 1/2/3. **(G–I)** ROC curves of the prognostic signature for 1-, 3-, and 5-year in AML validation set -1/-2/-3.

Validation for predictive performance from external independent AML cohorts is required for a reliable signature that is established on limited datasets. Three AML validation sets measured using a microarray platform were used to test the signature. Patients were divided into high- and low-risk groups based on FSAscore. In validation set 1, patients in the high-risk group have significantly shorter OS than those in the low-risk group (*p* = 5e-03, Figure 5A). A similar decreased OS was found in validation set 2 (*p* = 8.9e-03, Figure 5B) and validation set 3 (*p* = 5.3e-03, Figure 5C). This demonstrated that the signature still performs well on both array-based and RNA sequencing platforms. As the FSAscore increased, the death rate increased accordingly (Figures 5D–F). Then, the ROC curve was used to assess the predictive robustness. The AUC values of 1-, 3-, and 5-year OS were over 0.6 (Figures 5G–I). These data suggested that the signature has moderate performance for OS prediction for AML patients.

### Association of ferroptosis subtype-related signature with TIME and therapy

**FIGURE 6:**
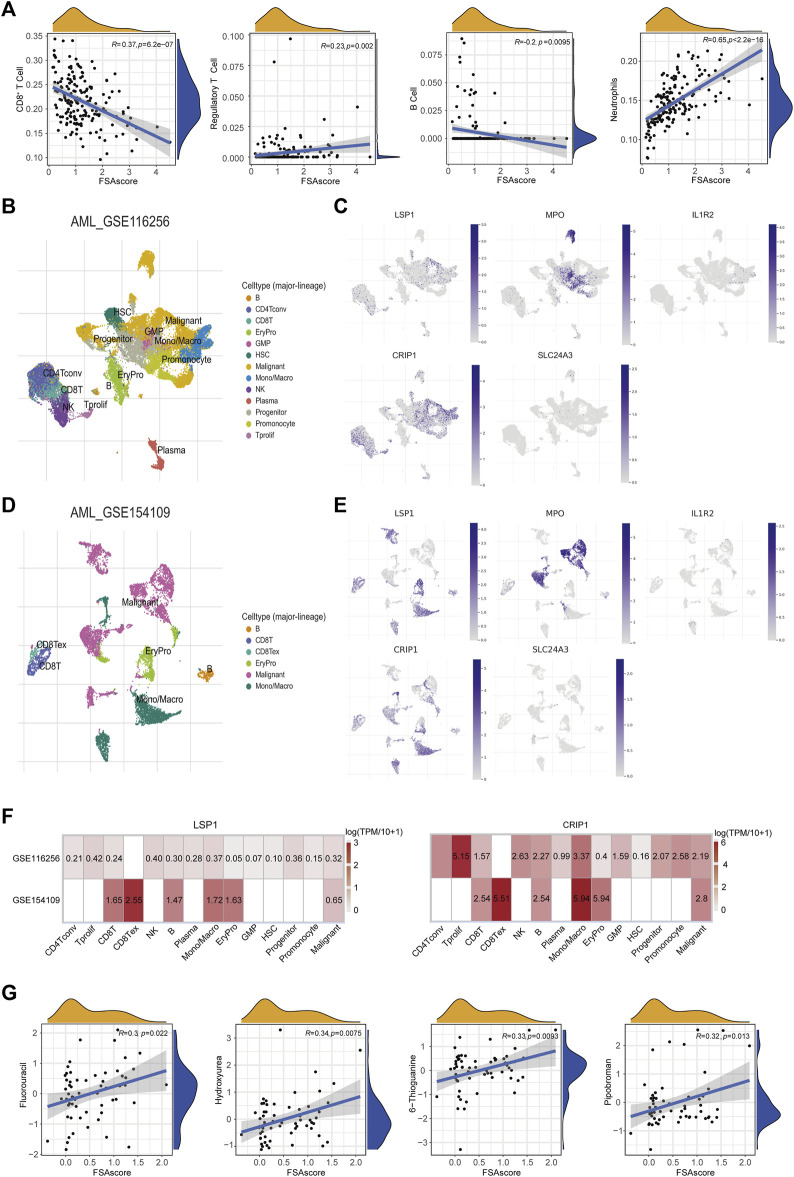
Association of the signature with TIME and drug sensitivity. **(A)** Pearson correlations of infiltrated CD8^+^ T cells, Tregs, B cells, and neutrophils with signature scores. **(B)** Cell clusters of AML (GSE116256) measured by single cell RNA-sequencing. **(C)** The expression levels of five signature genes in different cell clusters (GSE116256). **(D)** Cell clusters of AML (GSE1 54109) measured by single cell RNA-sequencing. **(E)** The expression levels of five signature genes in different cell clusters (GSE154109). **(F)** Heatmap of five signature genes expression in different cell clusters (GSE116256 and GSE154109). **(G)** Pearson correlations of GI50 of compounds under clinical trials or approved by the FDA with signature scores.

To investigate whether the ferroptosis subtype-related signature could characterize the TIME for AML patients, immune cell subsets were deconvoluted from the TCGA-AML dataset. We found that FSAscore was negatively correlated with the infiltration of CD8^+^ T cells and B cells and positively correlated with regulatory T cells and neutrophils (Figure 6A). This suggested that patients in the high-risk group exhibited suppressive TIME compared to those in the low-risk group, which might lead to a better prognosis in patients with low FSAscore, representing the immune phenotype of ferroptosis molecule-based classification. Accumulating evidence has supported the notion that TIME is closely correlated with treatment response. We used the library of compound sensitivity developed by DTP at NIH using 60 tumor cell lines to identify the sensitivity of relevant compounds of the ferroptosis-defined nature of the cell lines. The differential expression levels of five genes used to construct the signature were observed in the NCI-60 tumor cell line. LSP1 was highly expressed in most leukemia cell lines (shown in the dotted box, Supplementary Figure S3C), while IL1R2, MPO, and CRIP1 were highly expressed in one or two leukemia cell lines. SLC24A3 expression was quite low compared to the expression of the aforementioned four genes. In addition, their expression levels were upregulated in AML compared to those of normal cells (Supplementary Figure S3D). To further investigate the expression of these genes at the single-cell level derived from the AML microenvironment, we found that the expression of LSP1, MPO, and CRIP1 was higher in malignant cells in the GSE116256 dataset, which were consistent with aforementioned findings and previous reports (Zhao et al., 2018; Hosseini et al., 2019; Ma et al., 2020), while IL1R2 and SLC24A3 exhibited an extremely low level (Figures 6B, C). This was validated in an independent dataset (GSE154109, Figures 6D, E). Interestingly, LSP1 and CRIP1 were also expressed highly in immune cell subsets, including CD8^+^ T cells, exhausted CD8^+^ T cells, and mono/macrophages (Figures 6B–F), suggesting that targeting LSP1 and CRIP1 for AML might present a promising strategy. FSAscore was calculated for each cell line including leukemia cell lines. The correlation analysis of FSAscore with the concentration for 50% of maximal inhibition of compounds showed that 11 compounds under clinical trials or approved by the FDA were significantly positively correlated (Figure 6G; Supplementary Table S2). The evaluation of these compounds for their mechanisms of action (MOA) suggested that 4 of 11 compounds were alkylating agents, particularly those at the N-7 position of guanine showed significance, and tumor cell lines with high FSAscore were predicted to be more sensitive to these drugs.

## Discussion

Therapies with high efficacy and less toxicity for AML treatment are still under clinical trials. The low 5-year survival and high relapse rate of AML pose an urgent need to identify new prognostic biomarkers or develop effective therapies. Most effective antitumor treatment strategies are pursued to selectively eradicate cancer cells by inducing minimized damage to normal tissues or cells (Chen et al., 2021). Cell deaths are regulated or programmed by various forms of mechanisms, including apoptosis, necrosis, pyroptosis, autophagy, and autosis. Biological homeostasis and disease progression are usually controlled by different types of cell deaths (D'Arcy, 2019). Ferroptosis is a distinct form of cell death characterized by iron dependency and lipid peroxidation. An increasing number of studies have indicated that ferroptosis plays a dual role in tumorigenesis and the efficacy of anticancer therapeutics (Zuo et al., 2020; Chen et al., 2021). Genes that directly or indirectly drive or inhibit ferroptosis have been identified and have attracted significant attention recently. Ali Ghoochani et al. (2021) reported that ferroptosis inducers erastin or RSL3 could function independently or in combination with standard of care second-generation antiandrogens to inhibit cancer cell growth and migration *in vitro* and tumor growth *in vivo*, suggesting that induction of ferroptosis represents a promising therapeutic strategy for advanced prostate cancer. Cytotoxic T lymphocytes (CTLs), such as CD8^+^ T cells with potent activation and functions, are critical to kill leukemia cells, while most of these cells are inclined to exhibit an exhausted phenotype, leading to leukemia growth in the tumor niche. In addition, increased regulatory T-cell infiltration in bone marrow during disease progression suppresses anti-leukemia activities. Wang et al. (2019) demonstrated that ferroptosis is mediated by CD8^+^ T cells, which in turn influences the efficacy of immune checkpoint blockade. The role of ferroptosis in AML remains fully unexplored. Patients benefited from novel therapies including bispecific antibodies or molecular targeted therapies, while the long-term remission rate was quite low. Understanding ferroptosis within the AML immune microenvironment might have implications for identifying prognostic markers and developing novel therapies for AML.

Here, we demonstrated that the expression of FAGs is closely related to patient survival and TIME of AML. Two distinct molecular subtypes were identified by consensus clustering based on FAG expression. C1, with favorable clinical outcomes, was associated with enhanced infiltration of immune cells, especially CTL subsets that mainly function as tumor killers in the process of immune response such as CD8^+^ T cells. To characterize the immune landscape defined by the ferroptosis classification, we developed a 5-gene signature that showed performance efficiency in predicting prognosis, which was validated by multiple independent AML cohorts. This might provide a prognostic indicator for AML patients; however, it can be confirmed once it is verified in datasets from multi-centers and preferably even in clinical settings in the future. We predicted the potency of its association with TIME. The strong correlations of the signature with immune cells re-engraved the same trend of ferroptosis subtyping.

Ferroptosis has attracted significant attention in tumor biology and treatment strategy in recent years. The FAG expression in various solid tumors has been profiled, and many signatures based on the ferroptosis notion used to monitor outcomes and predict drug sensitivity have been proposed (Chang et al., 2021; Hong et al., 2021; Lu et al., 2021). Ruiming Qu et al. explored the association of ferroptosis-related genes with patient survival and found that increased expression of ARNTL in AML correlates with a poor prognosis (Yin et al., 2022). Comprehensive investigation of FAGs in AML is required because of the intensive genetic and epigenetic heterogeneity in the nature of AML (Li et al., 2016), and single genes might not capture the landscape. We noted that dozens of FAGs showed differential expression in AML compared to normal samples from the GTEx database. Some of those differentially expressed FAGs were linked with AML survival. In addition, pathway analysis showed that these genes are involved in cell death, metabolism, AML, and immune response. Among these survival-related FAGs, neutrophil cytosolic factor 2 (NCF2), a 67-kilodalton cytosolic subunit of the multi-protein NADPH oxidase (NOX) complex, was higher in AML and linked to decreased survival. NOX was one of the major contributors to reactive oxygen species (ROS) production (Valko et al., 2007). Low ROS release was correlated with the self-renewal of leukemia stem cells, and increased ROS promoted blast proliferation (Hole et al., 2013). NCF2 was also listed as one of the most upregulated genes in the unfavorable C2 subtype. Chemotherapeutic resistance is the major cause of treatment failure in AML. Paolillo et al. (2022) found that NOX genes, including NCF2, were elevated in AML cells that are resistant to daunorubicin or cytarabine and associated with immune signaling and inflammation. However, how NCF2 promotes AML aggressiveness and resistance was not specifically depicted. The role of NCF2 on immune cells in the TIME might be important, but it needs further research. Autophagy was also a dominant enriched pathway following ferroptosis, suggesting that these genes play multiple roles in cell death and partially share mechanisms of action in regulatory leukemia, which was also observed in our previously proposed autophagy-related signature that exhibits potent predictive performance for patient outcomes and immune landscape (Fu et al., 2021). This is consistent with emerging reports that ferroptosis is closely related to autophagy (Gao et al., 2016).

TME has been demonstrated to assess patient outcomes and response to therapies. Immune cell subsets were quantified using CIBERSORTx, TIMER, and xCell algorithms. Decreased CD8^+^ T cells and B cells were observed in the C2 subtype, while infiltrated suppressive subsets were increased, including neutrophils, macrophages, and Treg cells. Differential expression analysis found that many immune regulatory pathways are enriched that are representative of antigen processing and presentation, cytokine–cytokine receptor interaction, and B-cell receptor signaling. These results indicated hot immune activities in the C1 subtype. Accumulating studies have indicated that some crosstalk between autophagy and ferroptosis exists at the molecular level (Zhou et al., 2019). The infiltrating immune cell subsets stratified by our autophagy-related signature in AML in our previous analysis also showed similar results as the ferroptosis-based subtyping. Higher CD8^+^ T cells and lower Treg cells were observed in the low-risk group defined using an autophagy signature. Moreover, the correlations of these two types of cell death-related signatures with immune checkpoints showed overlap and difference similar to PD-1 (Fu et al., 2021). This may be induced by the complicated leukemia niche. We and other researchers have analyzed the TME of AML by scoring immune, stromal, and environments using the Estimation of STromal and Immune cells in MAlignant Tumor tissues using the Expression data (ESTIMATE) algorithm (Huang et al., 2019). TME-related genes were identified by comparing patients with high vs. low immune scores. Many of them also acted as the differentially expressed genes between C2 and C1, such as S100A8/9/12, CD163, CD86, IL1RN, CD1D, and NCF2. Their increased expression in the C2 subtype was associated with poor survival, which was also confirmed by comparing immune high vs. low AML groups in our previous publication (Huang et al., 2019). Accumulating evidence showed that S100A8 and S100A9 play pathogenic and prognostic roles in solid cancer types and hematological malignancies. Their expression is higher in AML, and patients with high S100A8 have poor prognosis (Laouedj et al., 2017; Mondet et al., 2021). S100A8 is reported to regulate autophagy-dependent ferroptosis in experimental subarachnoid hemorrhage (Tao et al., 2022), and S100A9 may also play regulatory roles in ferroptosis but lack “wet” experimental validation in head and neck squamous cell carcinoma (Liu et al., 2022). Increased KLF4 expression has been observed in AML and promotes disease progression (Morris et al., 2016). It can reverse polyphyllin Ⅲ-induced ferroptosis in triple-negative breast cancer by upregulation of xCT (Zhou et al., 2021), while its role in ferroptosis in AML has not been studied. The expression of LILRB3 and ITGB2 is also higher in AML, and LILRB3 acts as a marker for AML by modulating NF-κB signaling and promoting survival and immune evasion (Wu et al., 2021). LST1 is a regulator in inflammatory processes (Fabisik et al., 2021), and its role in AML is not defined. These findings indicated that data interpretation should be performed cautiously, and further work is warranted to reveal the roles of these genes in AML initiation and progression. Patients with liquid tumors benefited much from immune checkpoint inhibitors, while stable remission is still unsatisfactory (Pizzitola et al., 2014). We checked inhibitory immune checkpoint molecule expression and found that most of them were increased in the C2 subtype, suggesting that the patients in C2 might be sensitive to ICI-based therapies. This needs to be specified as some of these checkpoints could express on AML and immune cells. To further investigate the signatures that were used to calculate FSAscore in AML vs. normal cells, we found that all of them were increased in malignant cells using bulk RNA-seq data. As AML TME is complicated and associated with the outcome and treatment response, we investigated these signatures at a single-cell level and verified that they are higher in AML vs. non-malignant cells. In addition, some of them were also expressed on immune cells, such as cytotoxic T cells and NK cells, which are the main fighters against tumors. High LSP1 and CRIP1 levels correlate with unfavorable outcomes in our study, which is consistent with the previous evidence, suggesting they may serve as prognostic markers for AML patients. LSP1 and CRIP1 were elevated in exhausted CD8^+^ T cells, indicating that dual-targeting them may reverse the dysfunction of CD8^+^ T cells and inhibit leukemia growth to delay disease progression; however, further work is required to be carried out. In addition, the association analysis of the signature with NCI-60 tumor cell line growth inhibition screen data facilitated the identification of the potential compounds for AML. A total of 11 compounds that were under clinical or approved by the FDA for diverse tumors were screened, such as alkylating agents, particularly those at the N-7 position of guanine. Meanwhile, we should consider drug safety as it has been reported that they may increase the risk of AML, which needs to be confirmed in further experiments.

It is noteworthy that this study has potential limitations that inspire future validation and mechanistic studies. The molecular subtyping was established on the limited retrospective datasets; further validation in multi-centric AML sets may provide adjusted clues to make the classification to be more accurate. The utility of the proposed signature was not tested in a clinical setting, and quantification of immune subsets following applying the signature in AML patients will convince TIME predictive potential. Additionally, as CRIP1 and LSP1 are expressed on leukemia and cytotoxic immune cells, functional investigation of CRIP1 and LSP1 by AML cells or immune cells from TIME *in vitro* and *in vivo* assays will consolidate the dual-targeting treatment strategy for AML. Finally, we found that the expression of most immune checkpoint molecules was elevated in the unfavorable C2 subtype, indicating that patients with the C2 subtype may be sensitive to ICI-based therapy, while the association of our molecular subtyping with treatment response, especially immunotherapy, was not explored due to a lack of available datasets.

## Conclusion

This study depicted the molecular heterogeneity of AML through molecular subtyping by consensus clustering based on ferroptosis signatures. Two subtypes with distinct outcomes and TIME landscape were identified. Then, a 5-gene signature was constructed and validated, which could predict patient prognosis and characterize the ferroptosis subtype-related TIME. However, these data are derived from retrospective public datasets, and further investigation on the roles of FAGs in AML progression and assessment of the clinical use of the signature are imperative.

## Data Availability

The original contributions presented in the study are included in the article/Supplementary Material; further inquiries can be directed to the corresponding authors.

## Supplementary material

SUPPLEMENTARY FIGURE S1 Differentially expressed genes in AML vs. normal samples. **(A)** Principal component analysis of differentially expressed genes in AML vs. normal samples. **(B)** Top 10 differentially expressed ferroptosis-related genes ranked by the interaction degree.

SUPPLEMENTARY FIGURE S2 Forest plot showing overall survival-related differentially expressed genes between C2 vs. C1 subtypes (*p* < 0.05).

SUPPLEMENTARY FIGURE S3 Identification of key prognostic features via LASSO regression analysis. **(A)** 1,000-fold cross-validation for prognostic feature selection in LASSO regression analysis. **(B)** LASSO coefficients of key prognostic features. **(C)** The expression levels of genes that comprises of the signature in NCI-60 tumor cell lines. Dotted box represents the leukemia cell lines. **(D)** The expression levles of five signature genes in AML vs. normal samples.

SUPPLEMENTARY TABLE S1 Information of AML datasets used in this study.

SUPPLEMENTARY TABLE S2 Correlations of compound sensitivity of the NCI-60 tumor cell line with the signature.

## Abbreviations

AML, acute myeloid leukemia; FAGs, ferroptosis-associated genes; DEGs, differentially expressed genes; TME, tumor microenvironment; TIME, tumor immune microenvironment; CIBERSORT, cell-type identification by estimating relative subsets Of RNA transcripts; ICIs, immune checkpoint inhibitors; GEO, Gene Expression Omnibus; TCGA, The Cancer Genome Atlas; PD-1, programmed cell death 1; CTLA-4, cytotoxic T-lymphocyte-associated protein 4; GO, Gene Ontology; KEGG, Kyoto Encyclopedia of Genes and Genomes; OS, overall survival; ICI, immune checkpoint inhibitor; CTLs, cytotoxic T lymphocytes; ESTIMATE, Estimation of STromal and Immune cells in MAlignant Tumor tissues using Expression data; AIC, Akaike information criterion; ROC, receiver operating characteristic; AUC, area under the curve; GTEx, Genotype-Tissue Expression database; BP, biological process; CC, cellular component; MF, molecular function; HLA, human leukocyte antigen.
