# Supplementary figures and images for: Molecular subtyping of acute myeloid leukemia through ferroptosis signatures predicts prognosis and deciphers the immune microenvironment
Source: Front Cell Dev Biol. 2023 Aug 24;11:1207642. doi: 10.3389/fcell.2023.1207642 (PMC10483833; doi:10.3389/fcell.2023.1207642)

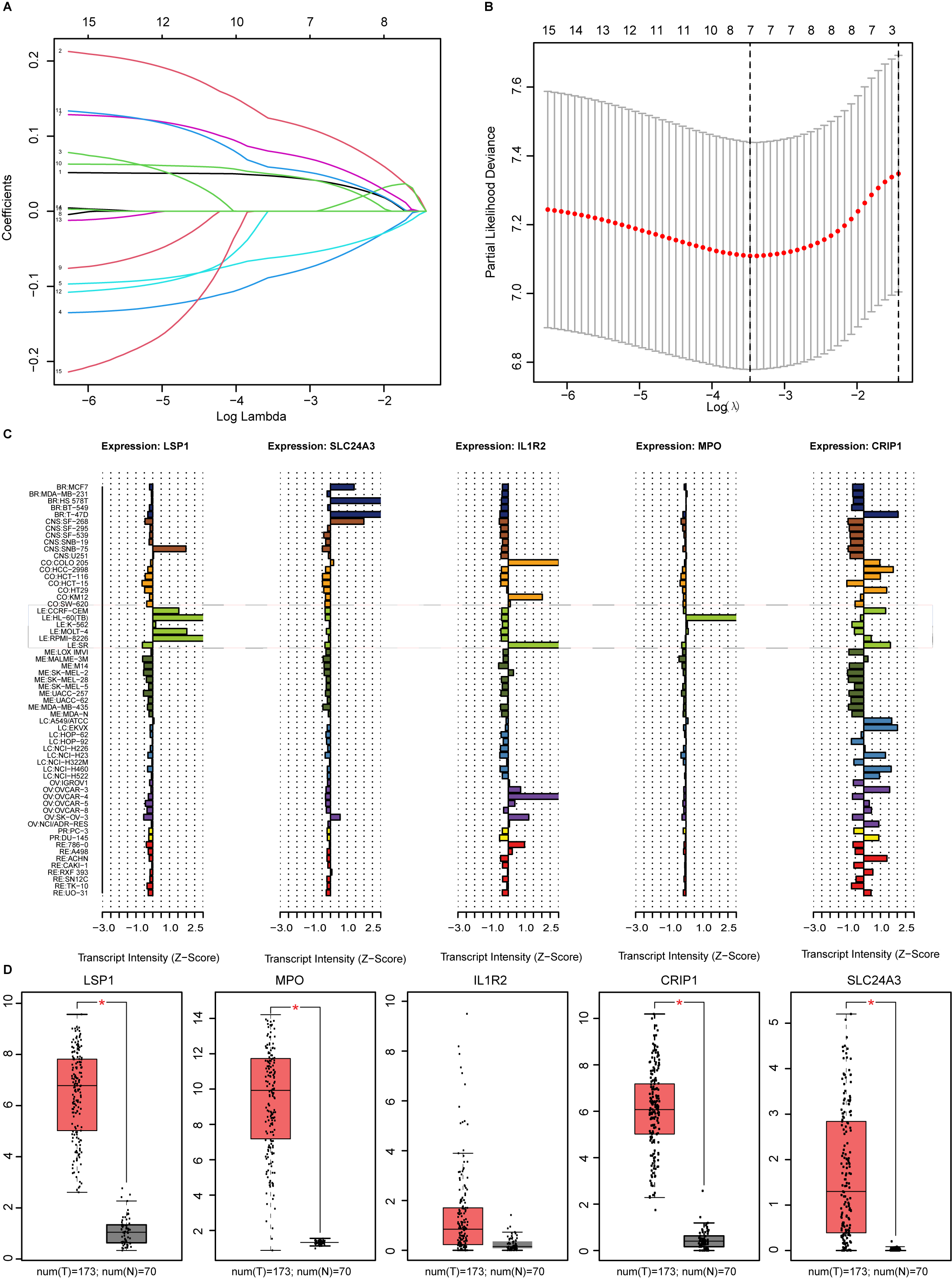

Supplement: Supplementary file 3 [file Image3.TIF]

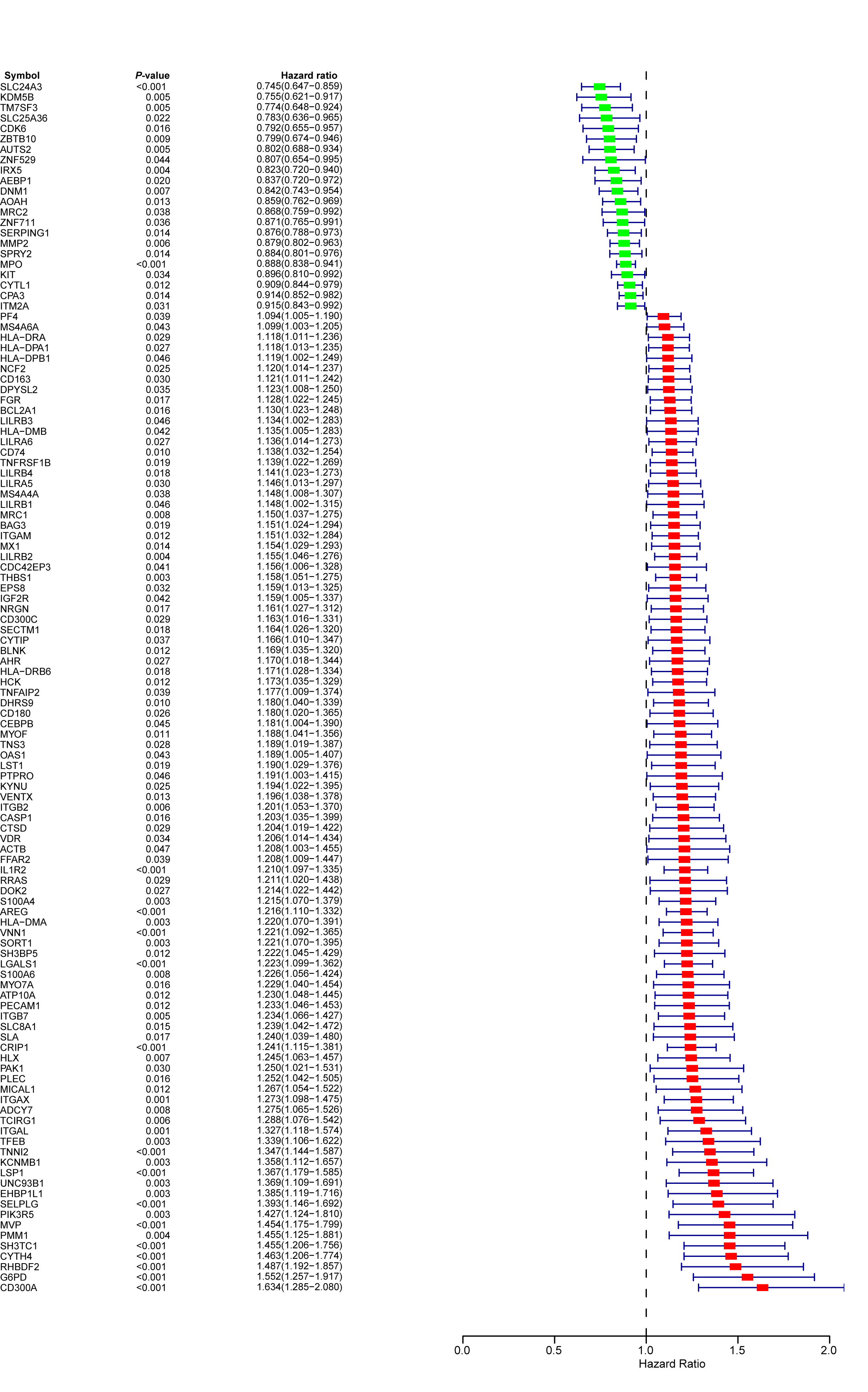

Supplement: Supplementary file 4 [file Image2.TIF]

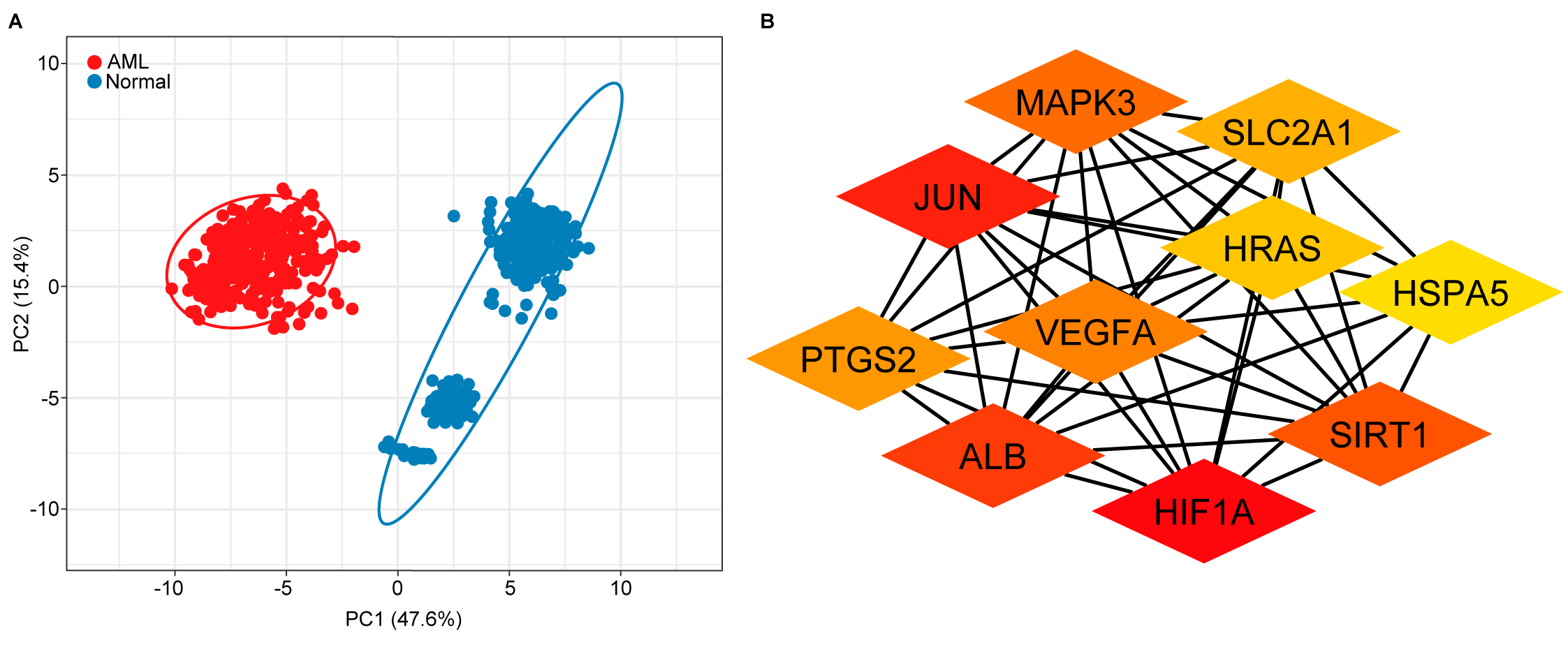

Supplement: Supplementary file 5 [file Image1.TIF]
